# The impact of environmental regulation on carbon emissions and its mechanisms in Chinese cities

**DOI:** 10.1038/s41598-025-25556-6

**Published:** 2025-11-24

**Authors:** Guichao Jin, Guoliang Wang, Wenjing Li, Haoxuan Sheng, Chunqi Hu, Yanfei Zhao

**Affiliations:** 1 Alibaba Business School, Hangzhou Normal University, Hangzhou, China; 2 School of Economics, Hangzhou Dianzi University, Hangzhou, China

**Keywords:** Environmental regulation, Carbon emissions, Technological progress, Foreign direct investment, Environmental sciences, Environmental social sciences

## Abstract

As the principles of green development continue to be implemented, China’s government-led and market-assisted environmental regulatory framework has become a crucial tool for driving continuous ecological improvement and advancing the construction of a Beautiful China. Under the “dual carbon” strategy, elucidating the mechanisms by which environmental regulation affects urban carbon emissions is of critical importance for facilitating green transformation and promoting high-quality development. Using panel data from 272 prefecture-level and above cities in China from 2003 to 2020, this study develops a composite index to quantify the intensity of urban environmental regulation. It systematically analyzes the spatial-temporal distribution patterns and evolutionary trends of both urban carbon emissions and regulatory intensity. Employing spatial autoregressive models and mediation effect models, the paper explores the impact of environmental regulation on carbon emissions and the underlying mechanisms at play. The key findings are as follows: First, both carbon emissions and the intensity of environmental regulation in Chinese cities show significant spatial clustering characteristics. Carbon emissions have shown an overall upward trend over the years, with notable regional disparities. The intensity of environmental regulation has generally fluctuated upward, although regional differences remain prominent. Second, environmental regulation significantly curbs carbon emissions, providing empirical support for the “forced emission reduction hypothesis”. Third, technological progress plays a partial mediating role in the relationship between environmental regulation and carbon emissions, thereby validating the “porter hypothesis”. Fourth, foreign direct investment also partially mediates the impact of environmental regulation on carbon emissions, lending support to the “pollution halo hypothesis”. The main contribution of this study lies in the systematic construction of a multidimensional mechanism framework for environmental regulation from a spatial perspective. It comprehensively identifies both the direct effects and indirect transmission pathways through which environmental regulation influences carbon emissions, thereby extending the theoretical boundaries of research on regulatory effects. Moreover, the findings provide a solid theoretical foundation and practical reference for the formulation of regionally differentiated carbon reduction policies.

**Supplementary Information:**

The online version contains supplementary material available at 10.1038/s41598-025-25556-6.

## Introduction

With the rapid pace of economic growth and accelerating industrialization, China’s energy consumption has continued to rise, resulting in a significant increase in total carbon emissions. This poses a significant policy challenge in achieving low-carbon and sustainable development both domestically and globally. At the same time, the coal industry remains a critical pillar for ensuring China’s energy security in both the present and foreseeable future^1^. According to the Statistical Bulletin of the People’s Republic of China 2022 National Economic and Social Development released by the National Bureau of Statistics in 2023^2^, the total annual energy consumption in 2022 reached 5.41 billion tons of standard coal, an increase of 2.9% over the previous year, accounting for about a quarter of the total global energy consumption. In the same year, the International Energy Agency’s “Carbon Dioxide Emission Report 2022” reported that China’s carbon dioxide emissions in 2022 totaled 11.477 billion tons, accounting for approximately one-third of global carbon emissions^3^. As such, China is confronted with dual challenges of accelerating the transformation of its energy consumption structure and fulfilling its obligations as a key stakeholder in global carbon mitigation initiatives. Effectively advancing carbon mitigation is therefore strategically crucial for promoting low-carbon and sustainable development both in China and globally^4^. It is important to emphasize that the primary sources and governance pressures of carbon emissions are highly concentrated in cities. As the core arenas of energy consumption, industrial production, transportation, and population agglomeration, cities account for more than 70% of China’s total carbon emissions and thus occupy an irreplaceable strategic position in achieving the goals of carbon peaking and carbon neutrality. Distinct emission patterns exist across different types of cities: resource-based cities in northern China, due to their heavy reliance on coal, have persistently maintained high emission intensities, whereas large coastal metropolitan areas face complex trade-offs among industrial upgrading, foreign investment inflows, and environmental quality improvement. At the same time, cities are interconnected through mechanisms such as policy competition, industrial relocation, and technological diffusion, which in turn generate significant spatial spillover effects and pronounced regional heterogeneity in carbon emissions. These characteristics indicate that research confined to the national or provincial level often obscures critical urban dynamics and fails to reveal the true mechanisms of policy transmission, regional interactions, and emission evolution. By contrast, adopting a city-level perspective not only deepens the understanding of the mechanisms and regional heterogeneity of environmental regulation but also provides a more solid scientific basis for designing context-specific and effective low-carbon policies.

In this context, achieving peak carbon emissions and subsequently reducing emission levels has become an imperative for China’s green development agenda and the realization of the “Beautiful China” initiative. The report of the 20th National Congress of the Communist Party of China emphasized the need to foster green production, promote fundamental improvements in the ecological environment, and drive high-quality development. In response to the pressing challenge of carbon emissions, the academic community has actively explored the application of advanced technologies such as deep learning to enhance the prediction and management of carbon emissions, thereby supporting the achievement of carbon neutrality goals^5–7^. Meanwhile, the Chinese government has explicitly set forth the “dual carbon” (carbon peaking and carbon neutrality) strategic goals and introduced a series of supporting policies. These include optimizing the energy mix, promoting clean energy, implementing energy-saving and emission-reduction actions, fostering low-carbon technological innovation, establishing a national carbon trading market, and advancing green finance initiatives. These efforts aim to achieve a synergistic balance between economic growth and environmental protection through the effective integration of governmental guidance and market mechanisms. It is worth noting that national-level strategic goals and policy initiatives can yield tangible emission-reduction outcomes only when effectively implemented at the urban level. Cities serve as critical arenas for policy enforcement, local governmental capacity, and the restructuring of industrial and technological systems. This reality further highlights the necessity and practical significance of investigating the relationship between environmental regulation and carbon emissions from an urban perspective.

Given the public goods nature of the natural environment and the negative externalities associated with carbon-emitting behaviors, relying solely on market forces to regulate carbon emissions often results in market failure, making it difficult to achieve effective emission reductions^8^. Therefore, environmental regulation through government intervention is essential to compensate for the shortcomings of market mechanisms and to provide robust support for achieving emission reduction targets^9^. Environmental regulation not only ensures the basic survival and development rights of society but also maximizes social welfare, serving as a crucial policy tool for promoting sustainable development^10^. Currently, there is no consensus within academic discourse regarding the impact of environmental regulation on carbon emissions. Research has primarily focused on both the direct and indirect effects. On the direct effect front, some scholars argue that environmental regulation induces a “forced emission reduction effect”, whereby stringent policies directly reduce pollutant emissions^11^. For example, Shapiro et al. (2018)^12^ found that since the implementation of the Clean Air Act, air pollutant emissions from the U.S. manufacturing sector have significantly declined. Guan et al. (2022)^13^, using a difference-in-differences model, found that China’s green performance assessment system significantly suppressed industrial pollutant emissions. Conversely, other scholars have proposed the “green paradox” hypothesis, suggesting that under local government competition pressures, stringent regulations may accelerate the extraction of fossil fuels to generate short-term economic gains or sustain local fiscal revenues, thereby increasing carbon emissions (Zhang et al., 2014; Wang et al., 2020)^14,15^. In addition, environmental regulation may indirectly affect carbon emissions through channels such as technological innovation and foreign direct investment. Regarding technological innovation, the “compliance cost theory” argues that environmental regulation raises pollution control costs for firms, thereby crowding out investment in innovation and impeding the achievement of environmental goals (Shi et al., 2018)^16^. In contrast, the “innovation compensation effect” posits that moderate environmental regulation can incentivize firms to increase R&D investment, promote innovation, and ultimately achieve pollution reduction (Peng et al.)^17^. In terms of foreign direct investment, the “pollution haven hypothesis” suggests that stringent environmental regulations may prompt firms to relocate pollution-intensive industries to regions with laxer regulatory standards to avoid higher compliance costs (Akar, 2019)^18^. However, the “pollution halo hypothesis” argues that along with the relocation of firms, advanced environmental technologies and higher operational standards may spill over positively and improve environmental quality in the host regions (Kisswani et al., 2021)^19^.

Although existing studies have provided valuable insights into the impact of environmental regulation on carbon emissions, several critical gaps remain. First, much of the current research adopts a unidimensional analytical perspective, lacking systematic modeling and rigorous identification of the multidimensional mechanisms through which environmental regulation influences carbon emissions via intermediary processes. Second, many studies rely predominantly on national or provincial-level data, limiting their capacity to capture spatial spillover effects and intercity policy interactions at the urban level. Third, there is a lack of adequate recognition of spatial heterogeneity in regulatory intensity and moderating mechanisms, which overlooks the crucial role of regional policy transmission pathways in shaping regulatory effectiveness. To address these limitations, this study makes three key contributions. First, it develops a multidimensional analytical framework that integrates direct effects, mediation mechanisms, and spatial spillover effects, enabling a more comprehensive understanding of the heterogeneous impacts of environmental regulation across various transmission channels. Second, the study constructs a panel dataset covering 272 prefecture-level and above cities in China from 2003 to 2020, allowing for a more fine-grained identification of regional disparities. Third, it applies spatial autoregressive models and spatial mediation models to investigate the interactive regional dynamics of environmental regulation, thereby enhancing the policy relevance and empirical robustness of the analysis. Compared with the existing literature, this paper offers three main marginal contributions in terms of theoretical development and methodological innovation. At the theoretical level, it incorporates both the green paradox and the compliance cost hypothesis into a unified analytical framework. By accounting for institutional characteristics such as local governments’ performance-driven incentives, energy dependency structures, and variations in environmental governance capacity, the study revisits the potential for regulation-induced preemptive resource exploitation, enriching localized interpretations of carbon emission rebound mechanisms. At the methodological level, the construction of a city-level spatial panel dataset provides greater granularity for identifying micro-level heterogeneity and improves the precision of spatial spillover effect estimation. In terms of model design, the study advances the spatial autoregressive approach by integrating a spatial mediation framework, which connects regulatory actions, intermediary processes, and emission outcomes, thereby contributing to a more systematic understanding of carbon reduction mechanisms.

## Research hypothesis

### The direct impact of environmental regulation on carbon emissions

Environmental regulation refers to the mandatory requirements imposed on firms or industries through laws, policies, and technical standards, with the aim of protecting the ecological environment and safeguarding public interests. In general, environmental regulation can be classified into market-based environmental regulation, administrative environmental regulation, and public-based environmental regulation. Yuan et al. (2014)^20^ further divided environmental regulation into formal environmental regulation and informal environmental regulation, and argued that administrative environmental regulation and market-based environmental regulation belong to formal environmental regulation, while spontaneous lobbying or negotiation activities initiated spontaneously by social groups or the public based on environmental awareness are considered informal environmental regulation. This paper primarily focuses on formal environmental regulation, specifically examining the joint impact of market-based and administrative environmental regulation on carbon emissions.

Due to its mandatory nature and government-led orientation, formal environmental regulation exerts a significant influence on carbon emissions. However, the direction of this influence remains ambiguous. Environmental regulation can lead to either a “reduction effect” or a “rebound effect” on emissions. On one hand, some scholars argue that environmental regulation produces a “forced emission reduction” effect^21^. This effect suggests that regulation increases the cost of environmental compliance for firms, compelling them to pursue technological innovation and industrial upgrading, thereby achieving energy conservation, emission reduction, and efficiency improvements. The mechanisms through which formal regulation reduces carbon emissions are threefold: First, government-led regulatory measures can eliminate outdated production capacity and force energy-intensive industries to upgrade and transform, thereby contributing to emission reductions^22^. Second, formal environmental regulation can reduce energy consumption by imposing environmental taxes or directly shutting down high-energy-consuming firms, thereby promoting a reduction in carbon emissions. Third, stringent environmental regulations encourage firms to adopt innovative methods, such as digital technologies, to avoid substantial fines, thereby achieving the goal of emission reduction^23^. On the other hand, some scholars point to the “green paradox” hypothesis, which posits that environmental regulation may paradoxically accelerate carbon emissions. Sinn (2008)^24^ argues that delaying the implementation of climate policies can create expectations among fossil fuel suppliers regarding future regulatory tightening. This anticipation may induce suppliers to expedite extraction and production of fossil resources to maximize short-term profits before stricter regulations take effect. Such behavior leads to an increase in fossil fuel supply and subsequently lowers energy prices (Zhang et al., 2014)^14^. The resulting decline in energy prices stimulates demand for fossil energy, thereby increasing carbon emissions and exacerbating environmental degradation.

Based on the above theoretical analysis, this study proposes the following hypotheses:

**H**_**11**_: **The implementation of environmental regulation may trigger a “green paradox effect”**,** resulting in an increase in total carbon emissions.**

**H**_**12**_: **The implementation of environmental regulation may generate a “forced emission reduction effect”**,** contributing to an effective reduction in total carbon emissions.**

### The indirect impact of environmental regulation on carbon emissions

Against the backdrop of rapid urban development and ongoing industrialization in China, environmental regulation not only affects carbon emissions through direct pollution control measures, but also exerts a profound indirect influence via technological progress in firms. Technological advancement within firms typically follows two main pathways: First, technological innovation, which refers to the enhancement of green innovation capabilities and core competitiveness through internal R&D efforts; Second, technology adoption, which relies on foreign direct investment and international technology transfer to import, absorb, and apply advanced low-carbon technologies, thereby elevating the overall level of green development in urban areas. In practice, the effectiveness of environmental regulation in stimulating these technological responses varies significantly across cities, due to differences in economic foundations, resource endowments, and policy enforcement capacities. These variations directly influence firms’ preferences and responsiveness when choosing between innovation and technology adoption in response to regulatory pressures, ultimately shaping the indirect pathways through which environmental regulation impacts carbon emissions.

(1) The “compliance cost hypothesis” and the “porter hypothesis”. The impact of environmental regulation on firms’ technological innovation is twofold and can be interpreted through the lenses of the “compliance cost hypothesis” and the “Porter hypothesis.” On the one hand, the compliance cost hypothesis posits that poorly designed, rigidly enforced, or excessively stringent environmental regulations compel firms to divert substantial short-term resources toward pollution control to meet compliance standards, thereby crowding out investment in green innovation (Gray & Shadbegian, 2003)^25^. This “cost-crowding effect” suppresses the process of technological upgrading, leading to a form of “negative technological lock-in”. Ultimately, under the influence of a path-dependent deterioration in technology, it indirectly drives up urban carbon emissions^26,27^. On the other hand, the Porter hypothesis argues that well-designed, moderate, and predictable environmental regulation can serve as a policy stimulus, encouraging firms to intensify R&D investment in clean production processes, energy efficiency, green technologies, and environmentally conscious management practices^28^. By enhancing technological capabilities and improving energy efficiency, firms are able not only to reduce their energy consumption but also to offset environmental compliance costs, thereby strengthening their competitive advantage in the market^29,30^. Through this positive mediating effect of technological upgrading, firms can effectively control carbon emissions while advancing sustainable development. In sum, environmental regulation indirectly determines urban carbon emission levels by influencing firms’ technological innovation behavior through a dual mechanism of cost-induced constraints and innovation-driven compensation. Based on the above theoretical analysis, this study proposes the following hypotheses:

**H**_**21**_: **Irrational environmental regulation suppresses technological upgrading and indirectly increases urban carbon emissions through the negative mediating effect of technological innovation**,** consistent with the compliance cost hypothesis.**

**H**_**22**_: **Rational environmental regulation stimulates technological upgrading and indirectly reduces urban carbon emissions through the positive mediating effect of technological innovation**,** consistent with the porter hypothesis.**

(2) The “pollution haven hypothesis” and the “pollution halo hypothesis”. A growing strand of research embeds spatial econometric techniques into analyses of urban carbon emissions and environmental regulation, emphasizing spatial dependence, spillovers, and policy interactions across cities. For instance, drawing on city-level panel data, Chen et al. (2022)^31^ and Liao et al. (2024)^32^ investigate the spatial spillover effects of China’s Low-Carbon City Pilot (LCCP) and the national carbon-emissions trading pilot (CETP). Their findings reveal that regulatory impacts extend beyond local boundaries and propagate to neighboring jurisdictions through intercity linkages. These results highlight the importance of incorporating cross-border policy externalities into assessments of environmental regulation effectiveness. In exploring the relationship between environmental regulation and carbon emissions, the academic community has developed two competing theoretical frameworks: the pollution haven hypothesis and the pollution halo hypothesis. These hypotheses offer distinct perspectives on how foreign direct investment and technology transfer influence carbon emissions. On the one hand, the Pollution Haven Hypothesis posits that pollution-intensive firms tend to relocate to regions with relatively lax environmental regulations^33^. Specifically, when the environmental standards in the host region are lower than those in the origin region, firms with high-pollution, energy-intensive, and high-emission may relocate to areas with more lenient pollution regulations in order to circumvent the stricter pollution control costs. This relocation process contributes to increased carbon emissions in the host cities. In the context of Chinese urban development, lenient environmental regulation may attract pollution-intensive firms, thereby exacerbating local carbon emissions. On the other hand, the pollution halo hypothesis offers a contrasting viewpoint. According to this hypothesis, during the process of foreign direct investment and technology transfer, host regions can benefit from the advanced environmental technologies and management practices employed by firms from more stringently regulated countries (Copeland & Taylor, 2001)^34^. Foreign direct investment not only brings capital but may also promote the green development of local firms by introducing green technologies, thereby reducing carbon emissions^35^. In the context of China’s pursuit of high-quality development, as environmental regulations are strengthened, foreign investment may encourage local firms to adopt advanced pollution control technologies. Through technology transfer and the dissemination of management experience, this can lead to a significant reduction in carbon emissions.

Therefore, the key distinction between the two hypotheses lies in the technological impact brought about by foreign direct investment. The entry of pollution-intensive firms directly increases carbon emissions, while the relocation of green development-oriented firms may indirectly drive the reduction of carbon emissions. This highlights the different mechanisms through which foreign investment affects carbon emissions in varying contexts. Based on the above theoretical considerations, this study proposes the following hypotheses:

**H**_**31**_: **Environmental regulation may increase total carbon emissions through the mediating effect of foreign direct investment**,** consistent with the pollution haven hypothesis.**

**H**_**32**_: **Environmental regulation may reduce total carbon emissions through the mediating effect of foreign direct investment**,** consistent with the pollution halo hypothesis.**

## Research design

### Variable selection and description

(1) Dependent variable. Currently, the predominant approaches to measuring carbon emissions are based on the application of carbon emission factors. While data calculated by official agencies-such as the National Bureau of Statistics-are relatively reliable at the provincial level, they often lack continuity and completeness at the prefecture-level city scale. In contrast, third-party institutions such as the Institute of Public & Environmental Affairs (IPE) and the China Emission Accounts and Datasets (CEADs) collect, verify, and calculate carbon emissions based on official statistics, resulting in more authoritative and continuous datasets. Given the considerations of data availability and continuity, this study measures city-level carbon emissions in China using data from the Institute of Public and Environmental Affairs (IPE). The IPE database is widely recognized for its credibility in the field of carbon accounting in China. It estimates annual carbon emissions at the city level by integrating enterprise-reported pollutant discharge data, publicly disclosed information from local governments, and standardized emission factor calculations. Compared with the commonly used China Emission Accounts and Datasets (CEADs), the IPE database provides broader spatial coverage and more frequent updates at the prefecture level and above, making it particularly suitable for spatial panel data analysis. To enhance the robustness and credibility of the dataset, this study also performs a cross-validation using CEADs data for selected regions. The results reveal consistent trends between the two data sources, suggesting that the IPE data are both representative and methodologically appropriate for the empirical analysis conducted in this research.

(2) Core independent variable. The existing literature presents four primary methods for measuring environmental regulation: ① Single-Indicator Method: This approach uses either the pollution control outcomes or costs of pollution control as proxies for the intensity of environmental regulation. While this method benefits from ease of data access and objectivity, it often provides a one-dimensional perspective and may overlook broader regulatory dynamics. ② Proxy Indicator Method: This method selects indicators that are highly correlated with regulatory intensity, yet statistically independent of pollution emissions themselves. While such indicators are generally available and continuous, they are susceptible to endogeneity issues. ③ Expert Scoring Method: Based on expert assessments, this approach assigns values to reflect the strength of environmental regulation. Although it can account for different types of regulation, it is highly subjective and may lack empirical robustness. ④ Composite Index Method: This method integrates multiple indicators of environmental regulation into a single composite index, thus addressing the limitations of single-dimensional approaches. It offers comprehensive and relatively objective measurements, although it requires large volumes of data, and access to consistent time-series data may be challenging.

From the perspective of measurement validity and data availability, this study employs the composite index method to quantify the level of environmental regulation with reference to the methodology proposed by Zhu et al.^36^. The relevant data are sourced from the China City Statistical Yearbook for the period 2004–2021. To ensure data continuity and accessibility, three indicators are selected to construct the environmental regulation index: industrial wastewater discharge, industrial soot and dust emissions, and industrial sulfur dioxide emissions^37^. The rationale for choosing these three indicators is as follows: ① These pollutants are among the most common emissions generated during industrial production processes and are capable of directly reflecting the intensity and effectiveness of environmental governance within the urban industrial sector. ② The corresponding indicator data exhibit strong continuity, completeness, and comparability across time and regions in the China City Statistical Yearbook, which is conducive to building a long-term, robust measurement system. ③ These pollutants are widely used in empirical research within environmental economics and environmental policy studies, making them typical and representative indicators for measuring regulatory effectiveness. Subsequently, the study applies the panel entropy method to calculate the composite index of environmental regulation. The specific computational procedure is outlined as follows:

Let *r* represent the number of years, *n* represent the number of regions, and *m* represent the number of indicators. Let *x*_*ijk*_ denote the value of the *k*-th indicator for the *j*-th region in the *i*-th year.

① The first step is data nor${M_{ij}}=\sum\nolimits_{k} {{w_k}} x_{{ijk}}^{{''}}$malization. The calculation formula for positive indicators is as follows:

1 $$x_{{ijk}}^{\prime }=\frac{{{x_{ijk}} - \hbox{min} {x_{ijk}}}}{{\hbox{max}{x_{ijk}} - \hbox{min}{x_{ijk}}}}$$

The calculation formula for negative indicators is: $x_{{ijk}}^{\prime }=\frac{{\hbox{max} {x_{ijk}} - {x_{ijk}}}}{{\hbox{max} {x_{ijk}} - \hbox{min} {x_{ijk}}}}$. max *x*_*ijk*_ and min *x*_*ijk*_ represent the maximum and minimum values of the k-th indicator across n regions and r years. After standardization, the standardized indicator $x_{{ijk}}^{\prime }$has a value range of [0, 1], which reflects the relative magnitude of *x*_*ijk*_ across n regions and r years. Since normalization may result in zero values, the data is subsequently shifted, yielding:

2 $$x_{{ijk}}^{{''}}=x_{{ijk}}^{\prime }+1$$

② The second step is to calculate the weight of the k-th indicator, denoted as *p*_*ijk*_:

3 $${p_{ijk}}=\frac{{x_{{ijk}}^{{''}}}}{{\sum\nolimits_{i} {\sum\nolimits_{j} {x_{{ijk}}^{{''}}} } }}$$

③ The third step is to calculate the entropy value of the k-th indicator, denoted as *e*_*k*_:

4 $${e_k}= - \frac{1}{{\ln (rn)}}\sum\nolimits_{i} {\sum\nolimits_{j} {{p_{ijk}}} } \ln ({p_{ijk}})$$

④ The fourth step is to calculate the coefficient of variation for the k-th indicator, denoted as *g*_*k*_:

5 $${g_k}=1 - {e_k}$$

⑤ The fifth step is to normalize the coefficient of variation and calculate the weight of the k-th indicator, denoted as *w*_*k*_:

6 $${w_k}=\frac{{{g_k}}}{{\sum\nolimits_{k} {{g_k}} }}$$

⑥ The final step is to calculate the composite index of environmental regulation, denoted as *M*_*ij*_:

7 $${M_{ij}} = \sum\nolimits_k {{w_k}} x_{ijk}^{''}$$

(3) Mediating variables. The pathways through which environmental regulation affects carbon emissions include technological innovation and foreign direct investment. Accordingly, this study selects two indicators to capture these mediation effects: the number of green invention patent applications filed in a given year^38^ and the actual amount of foreign direct investment^39^. The number of green patents effectively reflects the scale and intensity of environmentally oriented technological innovation at the regional level. As it is closely tied to environmental governance policies, this metric serves as a precise proxy for capturing the impact of environmental regulation on innovation activity. The actual amount of foreign direct investment, on the other hand, directly represents the capital flow behavior of foreign investors under the influence of environmental regulation, thereby offering a crucial indicator for assessing how foreign investment responds to regulatory intensity. Data on green invention patents are obtained from the China Patent Research Database, while foreign direct investment data are sourced from the China City Statistical Yearbook for the period 2004–2021.

(4) Control variables. Following standard practice in the existing literature^40–42^, this study incorporates the following control variables: economic development level, population size, industrial structure, fiscal capacity, capital investment, and labor input. The rationale for including these variables is as follows: ① Economic development level (measured by GDP) reflects a city’s foundational economic capacity and its ability to implement environmental governance. It is one of the core determinants of carbon emissions. ② Population size (measured by population density) directly influences the scale of urban production and consumption activities, which in turn affects environmental pressure and carbon output. ③ Industrial structure (measured by the share of secondary industry in GDP) significantly shapes the city’s industrial layout and environmental efficiency, thus capturing the influence of industrial upgrading and transformation on emissions. ④ Fiscal capacity (measured by the ratio of local general public budget expenditure to revenue) is a critical indicator of governmental financial support for urban environmental governance. It captures the extent to which local authorities can utilize discretionary fiscal resources and reflects their capacity for budgetary expansion, as well as the fiscal guarantees underpinning environmental regulatory implementation. In the context of China’s institutional framework, a fiscal pattern in which expenditures exceed revenues signals strong capabilities in fiscal coordination and resource mobilization. This enables local governments to allocate funds more flexibly toward ecological protection and green development, thereby strengthening the enforcement and effectiveness of environmental regulation^43^.⑤ Capital investment (measured by total fixed asset investment) and labor input (measured by the number of employees in urban units at year-end) represent the intensity of production factor inputs, which are core elements in production functions and widely used as control variables in economic analysis. All six control variables are drawn from the China City Statistical Yearbook covering the years 2004 to 2021. Detailed definitions and descriptions of the variables are presented in Table 1.

**Table 1:** Description of the variables.

Variable type	Variables	Symbols	Method of calculation
dependent variable	Carbon emissions	CE	Data provided by IPE
Core explanatory variables	Environmental regulation	ER	Composite index method
Mediating variables	Technological innovation	Tec	The number of green patents filed in the current year + 1
	Degree of openness	FDI	Actual foreign investment
Control variables	Economic level	GDP	Gross regional product
	Population size	Pop	Population density
	Industrial structure	Str	Output of the secondary industry/GDP
	Fiscal level	Fin	Expenditure of local general public budget/Revenue of local general public budget
	Capital input	Cap	Total fixed asset investment
	Labor input	Lab	Number of employed persons in urban establishments at the end of the period

### Model construction

The three most commonly used spatial econometric models in academic research are the Spatial Autoregressive Model (SAR), the Spatial Durbin Model (SDM), and the Spatial Error Model (SEM). Considering the significant spatial interaction effects in carbon emissions, this paper constructs the three spatial econometric models with reference to the methodology proposed by Li et al.^44^ and Wang et al.^45^. The specific models are presented as follows:

8 $$SAR:C{E_{it}}=\rho {W_{ij}}C{E_{it}}+{\beta _0}E{R_{it}}+{\beta _1}{X_{it}}+{d_i}+{u_t}+{\varepsilon _{it}}$$

 

9 $$SDM:C{E_{it}}=\rho {W_{ij}}C{E_{it}}+{\alpha _0}E{R_{it}}+{\alpha _1}{X_{it}}+{\nu _0}{W_{ij}}E{R_{it}}+{\nu _1}{W_{ij}}{X_{it}}+{d_i}+{u_t}+{\varepsilon _{it}}$$

10 $$SEM:C{E_{it}}={\delta _0}E{R_{it}}+{\delta _1}{X_{it}}+\lambda {W_{ij}}{\mu _{it}}+{d_i}+{u_t}+{\varepsilon _{it}}$$

Where, $C{E_{it}}$represents the carbon emission of the city *i* in the *t*-th year; $E{R_{it}}$represents the environmental regulation level of the city *i* in the *t*-th year; ${X_{it}}$represents the set of all control variables in this paper; ${W_{ij}}$represents the spatial matrix; $\rho$represents the spatial correlation coefficient of carbon emissions; $\lambda$represents the spatial error coefficient; $\nu$represents the spatial correlation coefficient of explanatory variables; ${d_i}$ represents city fixed effects; ${u_t}$represents year fixed effects. ${\mu _{it}}$ represents the spatial error term; ${\varepsilon _{it}}$ refers to the random error term.

## Results analysis

### Spatiotemporal evolution of environmental regulation

**Fig. 1:**
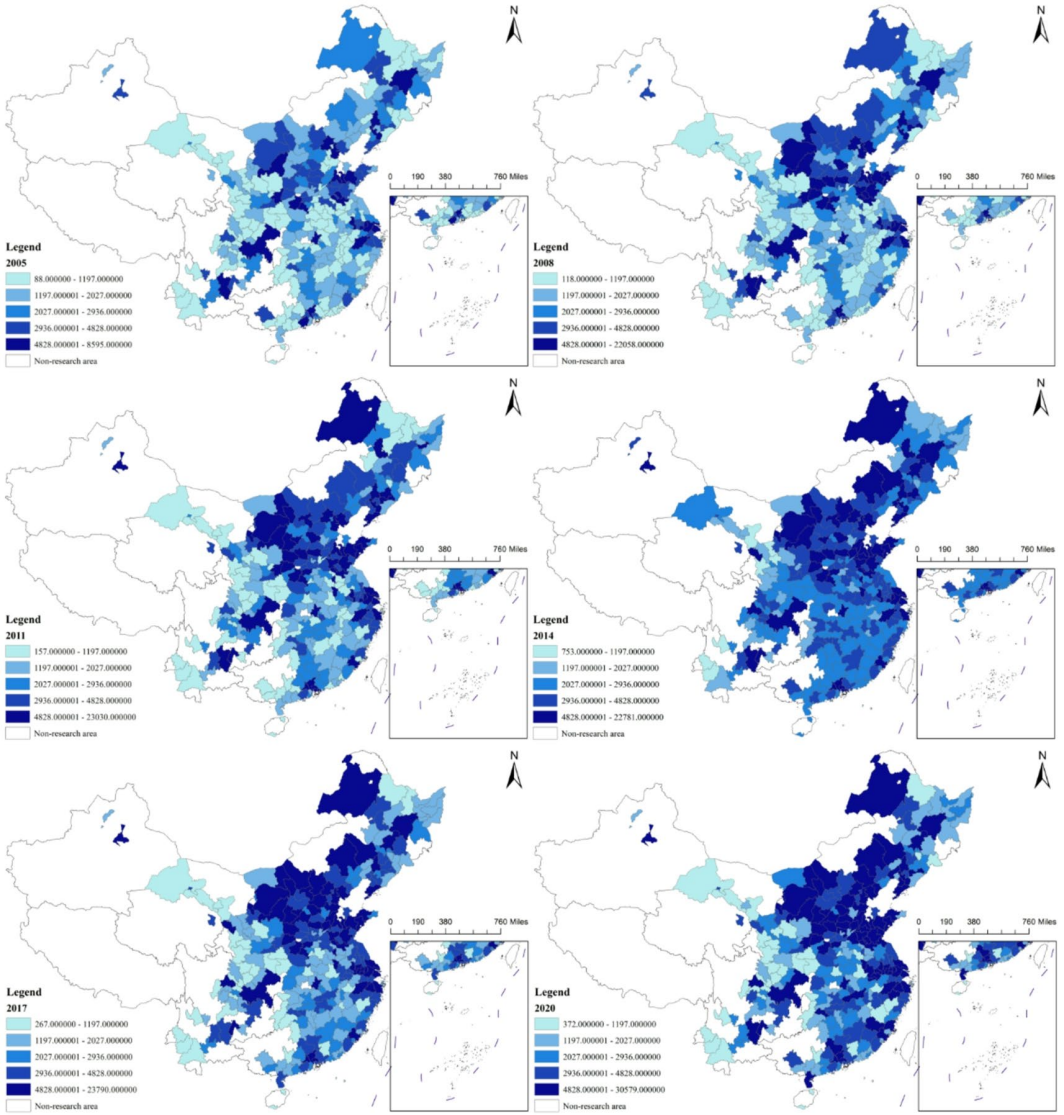
Spatiotemporal Evolution of Environmental Regulation Across Chinese Cities. Note: The map is based on the Standard Map GS (2020) No. 4618 from the Ministry of Natural Resources of China, with no modifications made to the base map boundaries.

This paper employs the entropy method to calculate environmental regulation data for a sample of 272 cities (excluding those with significant missing data) and utilizes ARCGIS software to produce spatiotemporal maps of environmental regulation for each city. Considering the impact of the COVID-19 pandemic, the study selects the years 2005, 2008, 2011, 2014, 2017, and 2019 as representative years. Using 2005 as the baseline, the natural breaks classification method provided by ARCGIS is applied to categorize the environmental regulation levels of each city into five tiers, from low to high. Darker colors indicate higher levels of environmental regulation in the corresponding cities. Figure 1 illustrates the level of environmental regulation in various cities across China.

From a temporal perspective, the environmental regulation levels in most cities show a fluctuating upward trend. This trend manifests in two main ways: First, starting from 2003, the environmental regulation levels of most cities gradually increased. However, in 2007 and 2008, there was a noticeable decline in the environmental regulation levels of many cities. This coincided with the global financial crisis, during which most cities faced a “dilemma” between economic development and environmental regulation, with the majority opting to prioritize economic growth. In 2008, the central government also introduced a 4-trillion-yuan investment plan to stimulate the economy. Second, starting from 2009, some cities gradually restored their environmental regulation levels, such as Chengdu, Guang’an, Qujing, and Urumqi, which began to strengthen their environmental regulations. Conversely, a portion of cities saw a gradual decline in their environmental regulation levels, such as Luoyang, Tangshan, Yingkou, and Changchun, where regulation levels decreased between 2008 and 2011. Third, from 2013 onward, with increasing national attention to ecological and environmental issues, as well as the introduction and implementation of the “Green mountains and clear water are as valuable as mountains of gold and silver” philosophy, environmental regulation levels in cities nationwide gradually improved to higher standards.

From a spatial distribution perspective, cities in northern China generally exhibit lower levels of environmental regulation compared to those in the south. This disparity can be attributed to several factors, including the fact that many northern cities are rich in mineral resources, and the extraction of these resources leads to significant pollution. In these cities, economic development is prioritized, often at the expense of lower levels of environmental regulations. Shanxi Province, for instance, is a typical example where cities generally have relatively low environmental regulation levels. The northeast region, due to its heavy industrial base, faces high pollution emissions, making it challenging to implement more stringent environmental regulations. In contrast, the northwestern region, particularly cities in Gansu Province, demonstrates relatively stronger environmental regulation. This may be attributed to the region’s less industrialized economic structure and the long-standing enforcement of stringent ecological and environmental policies in western China.

Regarding the spatial evolution trends, cities in the eastern region show a continuous increase in environmental regulation levels. As the eastern region pays more attention to the environment protection, high-pollution, high-energy-consuming industries have gradually shifted to less developed areas. From 2014 to 2017, cities in the southwestern region generally exhibited lower environmental regulation levels, potentially due to the transfer of industries from the eastern region and a deliberate relaxation of regulations to attract industries. From 2017 to 2019, cities in the central region, particularly those in Henan Province, saw a slight decline in environmental regulation levels.

### Spatiotemporal evolution of carbon emissions

**Fig. 2:**
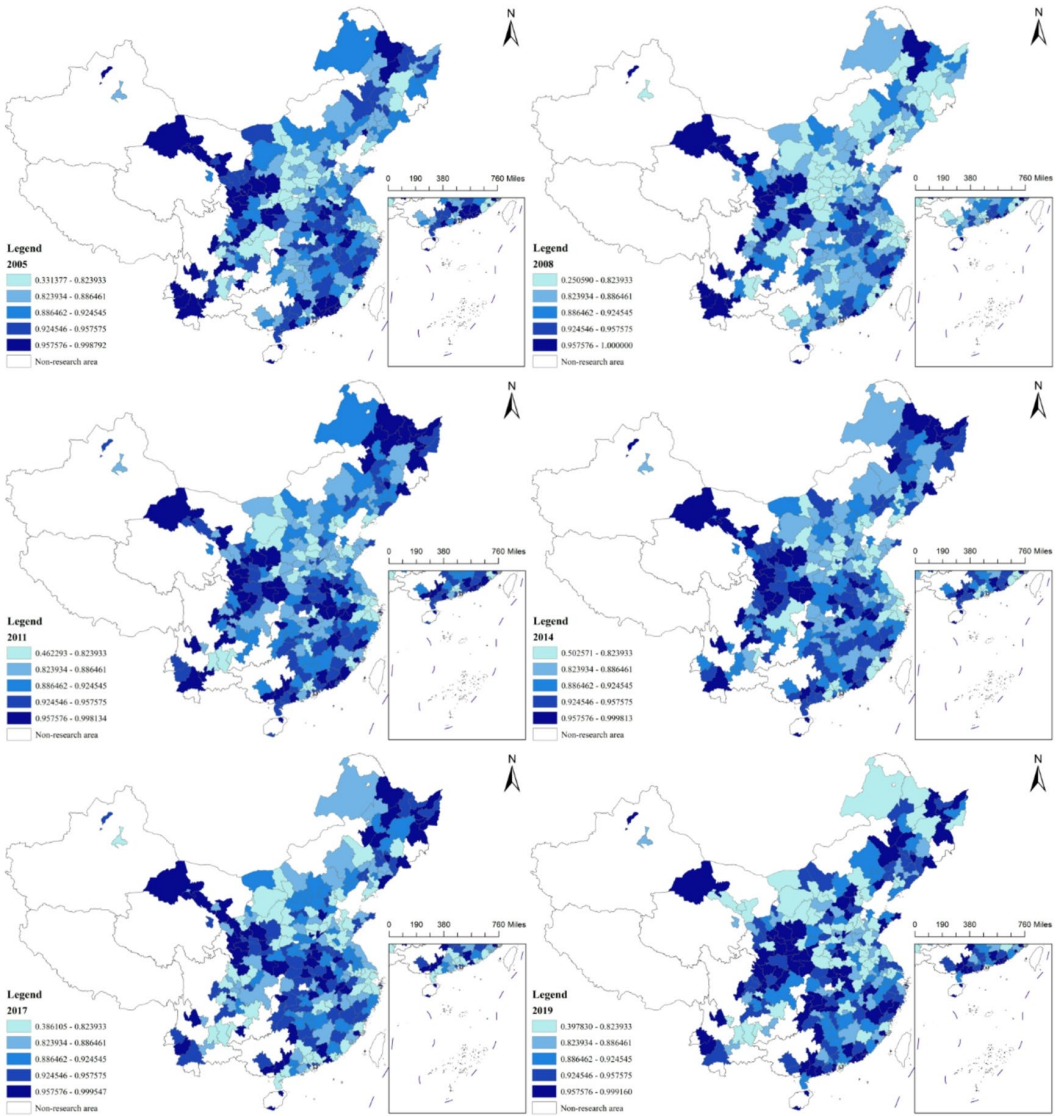
Spatiotemporal Evolution of Carbon Emissions Across Chinese Cities. Note: The map is based on the Standard Map GS (2020) No. 4618 from the Ministry of Natural Resources of China, with no modifications made to the base map boundaries.

This paper utilizes carbon emission data calculated by the IPE and imports it into ARCGIS software to create spatiotemporal maps of carbon emissions for the 272 city samples. Due to the large number of years involved, the paper selects 2005, 2008, 2011, 2014, 2017, and 2020 as representative years. Given that carbon emissions in earlier years were generally lower, 2011 is chosen as the baseline year. Using the natural breaks classification method provided by ARCGIS, the carbon emissions of each city are categorized into six tiers, from low to high. Darker colors indicate higher levels of carbon emissions in the corresponding cities. Figure 2 presents the carbon emission levels in various cities across China.

From a temporal perspective, the carbon emissions of cities in China generally show an upward trend over time. However, different time periods exhibit distinct characteristics. Firstly, since 2005, the carbon emissions of most cities have followed a continuous upward trajectory. However, a divergence emerged in 2011. A portion of cities experienced a continuous decline in carbon emissions, with this trend lasting until 2015, while emissions in another group of cities exhibited a year-on-year increase. Secondly, since 2015, there has been a trend of gradually decreasing carbon emissions in some cities. Although the carbon emissions of certain cities have shown a decline during specific periods, they still exhibit a significant increase compared to 2005. For instance, the carbon emissions in Shanwei increased by 1309% in 2018 compared to 2005; in Chaozhou, the emissions increased by 1178% in 2019, and in Ningde, the emissions rose by 848% in 2020. Notably, carbon emissions of Panzhihua have generally followed a downward trend year by year, with a rebound only occurring in 2018. Apart from Panzhihua, Zaozhuang is another example where the overall carbon dioxide emissions show a declining trend.

From a spatial distribution perspective, carbon emissions are generally higher in the North China, Northeast, and Western regions, while cities in the southern regions tend to have relatively lower emissions. At the city level, municipalities such as Beijing, Tianjin, Shanghai, and Chongqing exhibit higher carbon emission levels, with emissions exceeding 100 million tons in most years, and Shanghai surpassing 200 million tons. In addition to these municipalities, some prefecture-level cities also have high carbon emission levels, including Shijiazhuang, Tangshan, Handan, Linfen, Ordos, Nanjing, Suzhou, Wuxi, Hangzhou, Ningbo, Jinan, Weifang, Zibo, Jining, Wuhan, Yulin, and Yinchuan. Among these, Ordos and Tangshan have the highest carbon emissions among all prefecture-level cities. Carbon emissions in Ordos have been increasing steadily year by year, with emissions exceeding 200 million tons in both 2019 and 2020. Tangshan’s carbon emissions were around 100 million tons in the earlier years and have been steadily rising, surpassing 300 million tons by 2020, making it the highest among all prefecture-level cities.

### Spatial correlation analysis

This paper selects 272 city samples from 2003 to 2020 for descriptive statistics, and the statistical results are presented in Table 2.

**Table 2:** Descriptive statistical results.

Variable	Sample size	Mean	Standard deviation	Minimum value	Maximum value
CE	4896	7.717	0.866	4.477	10.419
ER	4896	−0.134	0.132	−1.464	0.000
GDP	4896	10.260	0.843	4.595	13.661
Fin	4896	0.870	0.538	−3.527	2.912
Lab	4896	4.041	2.269	1.399	16.252
Pop	4896	5.713	0.943	1.398	9.084
Str	4896	3.822	0.265	1.334	4.511
Cap	4896	15.690	1.229	12.018	19.837
Tec	4896	3.461	1.951	0.000	10.088
FDI	4896	9.994	1.911	0.693	15.997

Table 2 presents the descriptive statistics of the key variables. First, regarding carbon emissions (CE), the sample mean is 7.717 with a standard deviation of 0.866, a minimum value of 4.477, and a maximum value of 10.419. These figures indicate a moderate overall variation in carbon emission levels across regions, though the considerable range between the minimum and maximum values reflects substantial regional heterogeneity. This underscores the urgent need for stricter and more targeted emission reduction policies in high-emission areas, particularly in the context of promoting regionally coordinated low-carbon development. Second, the intensity of environmental regulation (ER) has a mean of −0.134, a standard deviation of 0.132, a minimum of −1.464, and a maximum of 0. These results suggest that environmental regulation is generally weak across the sampled regions, with notable spatial disparities. Such imbalances in regulatory strength may contribute to uneven regional performance in carbon governance and pose challenges to achieving nationwide emission reduction targets. In terms of technological innovation (Tec), the mean value is 3.461 with a standard deviation of 1.951, ranging from 0 to 10.088. This indicates a significant disparity in innovation capacity across regions. While some areas demonstrate strong capabilities in green technology research and development, others face serious constraints or even a lack of innovation infrastructure. These disparities may directly affect the efficiency of emission reduction efforts and the depth of green transformation. With respect to foreign direct investment (FDI), the mean is 9.994 and the standard deviation is 1.911, with values ranging from 0.693 to 15.997. This highlights pronounced differences in the ability of regions to attract foreign capital. On one hand, highly open regions may leverage foreign investment to promote green and low-carbon development through the introduction of advanced production technologies. On the other hand, in the absence of effective regulation, there is also a potential risk of pollution-intensive industries relocating under the “pollution haven” effect. Overall, the sampled regions exhibit substantial spatial heterogeneity in terms of carbon emission levels, regulatory intensity, innovation capacity, and FDI inflows. These disparities increase the complexity of coordinated regional carbon reduction governance and suggest that policy design must adhere to a principle of context-specificity. A balanced approach that simultaneously strengthens environmental regulation, enhances technological innovation, and improves mechanisms for guiding foreign investment is essential for achieving high-quality and sustainable low-carbon development.

(1) Space weight setting.

The weight of the inverse geographic distance matrix is defined as the spatial weight, which is based on the reciprocal of the distance. Specifically, the closer the distance between two locations, the greater the weight; conversely, the farther apart the locations are, the smaller the weight. In this context, *d*_*ij*_ represents the distance between location i and location j, while *W*_*ij*_ denotes the spatial weight between them.

11 $${W_{ij}}=\left\{ \begin{gathered} 0{\kern 1pt} {\kern 1pt} {\kern 1pt} {\kern 1pt} {\kern 1pt} {\kern 1pt} {\kern 1pt} {\kern 1pt} {\kern 1pt} {\kern 1pt} {\kern 1pt} {\kern 1pt} {\kern 1pt} {\kern 1pt} {\kern 1pt} {\kern 1pt} {\kern 1pt} {\kern 1pt} {\kern 1pt} {\kern 1pt} {\kern 1pt} {\kern 1pt} {\kern 1pt} {\kern 1pt} (i=j) \hfill \\ 1/\left| {{d_{ij}}} \right|{\kern 1pt} {\kern 1pt} {\kern 1pt} {\kern 1pt} {\kern 1pt} {\kern 1pt} (i \ne j) \hfill \\ \end{gathered} \right.$$

(2) Global spatial autocorrelation test.

Spatial autocorrelation refers to the phenomenon where neighboring regions exhibit similar values for a given variable. If high values cluster together and low values also cluster together, it is termed “positive spatial autocorrelation”; if high values and low values cluster together, it is referred to as “negative spatial autocorrelation”. In the case where high and low values are randomly distributed, spatial autocorrelation is absent.

The primary method for measuring spatial autocorrelation is the Moran’s Index (Moran’s I), which is further categorized into global and local Moran’s indexes. The formula for calculating the global Moran’s index is as follows:

12 $$I=\frac{{\sum\nolimits_{{i=1}}^{n} {\sum\nolimits_{{j=1}}^{n} {\left[ {{w_{ij}}({x_i} - \overline {x} )({x_j} - \overline {x} )} \right]} } }}{{{S^2}\sum\nolimits_{{i=1}}^{n} {\sum\nolimits_{{j=1}}^{n} {{w_{ij}}} } }}$$

Where, ${S^2}=\frac{{\sum\nolimits_{{i=1}}^{n} {{{({x_i} - \overline {x} )}^2}} }}{n}$is the sample variance, ${w_{ij}}$denotes the element in the i-th row and j-th column of the spatial weight matrix, ${x_i}$and${x_j}$ represent the variable values of different regions. $\overline {x}$ is the mean of the value, and$\sum\nolimits_{{i=1}}^{n} {\sum\nolimits_{{j=1}}^{n} {{w_{ij}}} }$ is the sum of all spatial weights. The value of Moran’s I generally ranges from − 1 to 1. A value greater than 0 indicates positive spatial autocorrelation, meaning high values are adjacent to high values and low values are adjacent to low values. A value less than 0 indicates negative spatial autocorrelation, meaning high values are adjacent to low values. A value of 0 indicates the absence of spatial autocorrelation. Table 3 presents the global Moran’s I for carbon emissions in cities from 2003 to 2020, calculated using the inverse distance spatial weight matrix.

**Table 3:** Global moran’s I for each year.

Year	Moran’s I	Year	Moran’s I	Year	Moran’s I
2003	0.0406^***^	2009	0.0577^***^	2015	0.0600^***^
2004	0.0487^***^	2010	0.0529^***^	2016	0.0669^***^
2005	0.0438^***^	2011	0.0591^***^	2017	0.0693^***^
2006	0.0508^***^	2012	0.0612^***^	2018	0.0715^***^
2007	0.0541^***^	2013	0.0661^***^	2019	0.0724^***^
2008	0.0552^***^	2014	0.0494^***^	2020	0.0697^***^

From Table 3, it can be observed that the Moran’s I for all years are positive, and the P-values all pass the significance test at the 1% level. This indicates a significant positive spatial autocorrelation in the carbon emissions of various cities. In other words, regions with high carbon emissions tend to be spatially clustered with other regions exhibiting similarly high emissions, while low carbon emission areas also demonstrate significant spatial clustering. Therefore, on a broader scale, the carbon emissions in Chinese cities show a clear spatial agglomeration characteristic.

(3) Local autocorrelation test.

The global autocorrelation results examine the overall spatial clustering of carbon emissions across the entire study area. However, to assess the spatial clustering in the vicinity of individual regions, the Local Moran’s I index is used. The formula for calculating the Local Moran’s I is as follows:

13 $${I_i}=\frac{{({x_i} - \overline {x} )}}{{{S^2}}}\sum\nolimits_{{j=1}}^{n} {{w_{ij}}} ({x_j} - \overline {x} )$$

If ${I_i}$ is positive, it indicates that the values of the neighboring regions are similar to those of region *i*, implying a high-high spatial clustering. If ${I_i}$ is negative, it suggests that the values of the neighboring regions are opposite to those of region *i*, indicating a low-high spatial clustering. Given the large number of results from the Local Moran’s Index, this paper selects Moran scatter plots of the years 2005, 2008, 2011, 2014, 2017, and 2020 for analysis.

**Fig. 3:**
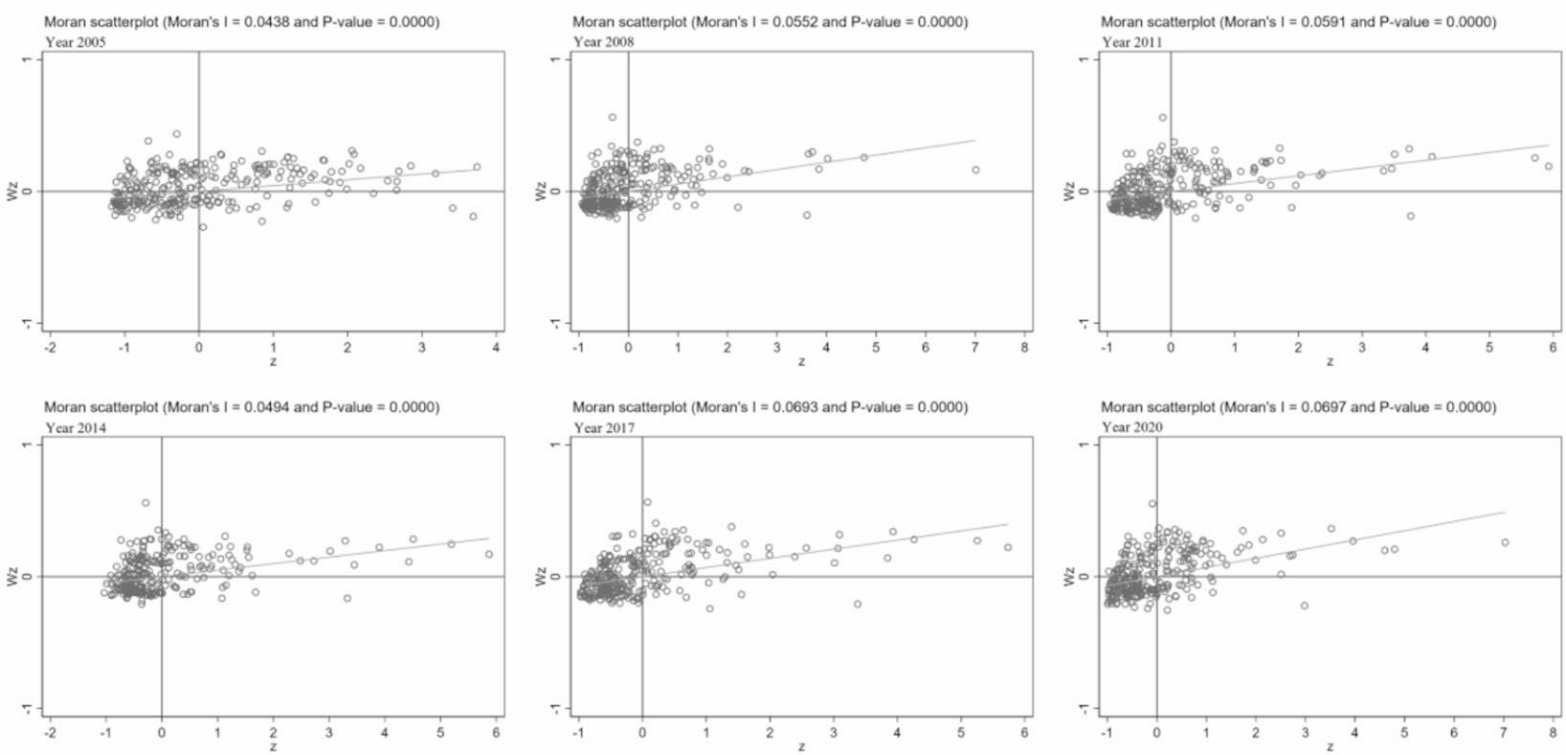
Moran scatter plots for representative years of carbon emissions across Chinese cities.

Figure 3 presents Moran scatter plots illustrating the distribution of carbon emissions across cities for selected representative years. The first quadrant represents high-high clustering of carbon emissions, the second quadrant indicates low-high clustering, the third quadrant shows low-low clustering, and the fourth quadrant reflects high-low clustering. It can be observed from the figure that most years fall within the first and third quadrants, although some cities are situated in the second and fourth quadrants. This suggests that the carbon emissions of most cities exhibit high-high and low-low clustering patterns, demonstrating a strong spatial correlation at the local level, which also validates the results of the global Moran’s I.

### Benchmark Estimation results

The results of the Hausman test indicate a chi-square statistic of 1181.20 with a p-value of 0.0000, suggesting a significant difference between the fixed effects and random effects models. A statistically significant p-value leads to the rejection of the null hypothesis (i.e., that the differences in coefficients are not systematic), thereby supporting the adoption of the fixed effects model. Considering that the fixed effects model effectively controls for individual heterogeneity and avoids potential bias introduced by the random effects model, a two-way fixed effects model is deemed more appropriate for this study. Moreover, in order to examine whether the Spatial Durbin Model (SDM) degenerates into either the Spatial Autoregressive Model (SAR) or the Spatial Error Model (SEM), this study conducted a series of likelihood ratio (LR) tests. The results reveal that the LR tests fail to reject the null hypothesis (all p-values equal to 1.000), indicating that the SDM may indeed degenerate into either the SAR or SEM models. Simultaneously, the results of the Lagrange Multiplier (LM) tests show that both the LM-lag and the Robust LM-lag tests reject the null hypothesis at the 1% significance level (both p-values are 0.000), further supporting the preference for the SAR model over the SDM. Based on this series of diagnostic tests, this study ultimately selects the Spatial Autoregressive Model (SAR) for empirical analysis. The baseline regression results are presented in Table 4.

**Table 4:** Benchmark regression Estimation Results.

Variable	(1) CE	(2) CE
ER	−0.272^***^ (−5.02)	−0.209^***^ (−3.91)
GDP		0.267^***^ (10.10)
Cap		−0.025 (−1.59)
Pop		−0.016 (−0.37)
Fin		−0.138^***^ (−5.22)
Str		−0.163^***^ (−4.23)
Lab		0.020^**^ (2.45)
$\rho$	2.318^***^ (79.08)	2.239^***^ (47.36)
City fixed effects	Yes	Yes
Year fixed effects	Yes	Yes
N	4896	4896
R^2^	0.064	0.239

Note: The values in parentheses represent the Z-statistics; *, **, and *** denote significance at the 10%, 5%, and 1% levels respectively, and the same below.

Columns (1) to (2) in Table 4 present the regression results for the impact of environmental regulation on carbon emissions with the inclusion of control variables. The results indicate that although the coefficient of environmental regulation on carbon emissions decreases from − 0.272 to −0.209 after adding a series of control variables, the effect remains statistically significant at the 1% level. This suggests that environmental regulation has a significant inhibitory effect on carbon emissions, meaning that a 1% increase in environmental regulation leads to a 0.209% reduction in carbon emissions. In view of this, Hypothesis H_12_ is validated, confirming that the effect of environmental regulation on carbon emissions aligns with the “forced emission reduction theory”. A possible explanation is that, in recent years, China has significantly tightened its approval procedures for fossil energy development. Under the constraints of the “dual carbon” targets, local governments have strengthened their control over preemptive extraction behavior, effectively weakening the practical relevance of the green paradox mechanism. Moreover, most cities have yet to establish a fully functioning carbon pricing system, which limits the ability of regulatory expectations to generate strong anticipatory constraints on fossil resource extraction. As a result, such expectations have not triggered widespread incentives for accelerated resource exploitation. These findings provide empirical support for the limited applicability of the green paradox mechanism at the urban level in China. Additionally, the spatial spillover coefficient, ρ, is significant at the 1% level, indicating that changes in the level of environmental regulation can have a positive spatial spillover effect on carbon emissions in neighboring cities. Regarding the control variables, changes in population density and capital investment do not significantly affect carbon emissions. However, changes in economic development level and labor input have a significant positive impact on carbon emissions, while changes in fiscal expenditure and industrial structure significantly reduce carbon emissions. Table 5 reports the results of the spatial effect decomposition, including both direct and indirect effects. With respect to the direct effects, the estimated coefficient of environmental regulation is significantly negative, indicating that local environmental regulation effectively reduces carbon emissions within the region. In contrast, the estimated coefficient for the spatial spillover effect (i.e., the indirect effect) is significantly positive, suggesting that while stringent environmental policies suppress emissions locally, they may inadvertently lead to increased carbon emissions in neighboring regions. Several mechanisms may account for this finding. First, driven by profit maximization and cost minimization motives, high-pollution and energy-intensive firms facing stringent local environmental regulations often relocate their pollution-intensive production processes to nearby areas with looser environmental standards and lower regulatory costs. This interregional relocation of pollution-intensive activities is consistent with the “pollution haven” hypothesis, whereby strict environmental policies in one region induce firms to spatially reallocate their operations, making neighboring regions de facto recipients of pollution spillovers. Second, in an effort to attract these displaced industries and production factors, neighboring regions may deliberately maintain lower environmental standards or adopt a more lenient regulatory posture. This regulatory “race to the bottom” further exacerbates interregional disparities in environmental enforcement intensity. Such a “policy trough effect” not only undermines the overall effectiveness of carbon reduction strategies but may also trigger environmental competition among regions, resulting in the externalization of pollution control and the internalization of pollution burdens. In addition, the direct effect of GDP is significantly positive, whereas the indirect effect is significantly negative. This indicates that local economic growth contributes directly to higher local carbon emissions, but also facilitates emission reductions in surrounding areas through channels such as technological diffusion, industrial upgrading, and economic interconnectivity. This finding highlights the positive spatial externalities of regional economic development: economic dynamism and technological advancement can promote improvements in production efficiency and energy utilization in adjacent regions via upstream and downstream industrial linkages, technology demonstration effects, and the mobility of human capital, thereby contributing to lower carbon emissions in neighboring areas.

**Table 5:** Spatial decomposition of the effects of environmental regulation on carbon Emissions.

	(1)	(2)	(3)
	Total Effect	Direct Effect	Indirect Effect
ER	−0.166^***^	−0.245^***^	0.078^***^
	(0.038)	(0.054)	(0.020)
Str	−0.132^***^	−0.194^***^	0.062^***^
	(0.028)	(0.039)	(0.015)
Fin	−0.088^***^	−0.129^***^	0.041^***^
	(0.018)	(0.025)	(0.010)
GDP	0.234^***^	0.344^***^	−0.111^***^
	(0.024)	(0.032)	(0.018)
Lab	0.166^***^	0.245^***^	−0.079^***^
	(0.057)	(0.083)	(0.030)
Pop	−0.172^***^	−0.253^***^	0.081^***^
	(0.059)	(0.087)	(0.031)
Cap	−0.046^***^	−0.068^***^	0.022^***^
	(0.012)	(0.018)	(0.007)
N	4896.000	4896.000	4896.000

### Robustness tests and endogeneity analysis

To further verify the reliability of the results, this paper conducts two robustness checks (Zhu, et al., 2023)^46^. First, the design of the spatial matrix could impact the results. Therefore, an adjacency matrix is used as a substitute for the inverse geographic matrix for analysis, with the results presented in column (1) of Table 6. Second, considering that municipalities directly under the central government (Beijing, Tianjin, Chongqing, and Shanghai) are characterized by more concentrated resources, larger populations, and stronger economic vitality compared to other cities, the data from these cities are excluded to enhance the comparability of the sample. The regression results after this exclusion are shown in column (2) of Table 6.

Drawing on the analytical framework proposed by Zhao et al. (2012)^47^, this study employs the interaction terms between the spatial weight matrix and the explanatory variables as instrumental variables and conducts systematic estimation using the two-stage least squares (2SLS) method. The regression results are presented in columns (3) and (4) of Table 6. In the first-stage regression, the KP-F statistic is 221.347, significantly exceeding the critical value at the 10% bias level, which shows that the selection of instrumental variables is reasonable and effective through weak instrumental variable test. The second-stage regression results show that even after mitigating potential endogeneity issues, environmental regulation still exerts a statistically significant suppressive effect on carbon emissions.

**Table 6:** Robustness tests and endogeneity Analysis.

Variable	(1) Adjacency matrix	(2) Data exclusion	(3) First Stage	(4) Second Stage
ER	−0.288^***^ (−4.86)	−0.228^***^ (−3.93)		−0.190** (−2.15)
IV			0.018*** (7.55)	
Gdp	0.287^***^ (10.04)	0.275^***^ (10.28)	−0.023** (−2.06)	0.263*** (8.84)
Cap	−0.007 (−0.41)	−0.028^*^ (−1.71)	−0.002 (−0.29)	−0.008 (−0.46)
Peo	−0.067 (−1.31)	−0.001 (−0.04)	−0.019* (−1.70)	0.052 (1.51)
Fin	−0.062^**^ (−2.06)	−0.133^***^ (−4.99)	0.014* (1.75)	−0.156*** (−5.51)
Str	−0.088^**^ (−2.05)	−0.176^***^ (−4.24)	0.027** (2.12)	−0.094** (−2.21)
Lab	0.013^**^ (2.06)	0.021^**^ (2.53)	−0.010 (−1.23)	0.083*** (5.19)
$\rho$	0.294^***^ (621.93)	2.264^***^ (47.07)		2.160*** (31.89)
City fixed effects	Yes	Yes	Yes	Yes
Year fixed effects	Yes	Yes	Yes	Yes
N	4896	4824	4896	4896
R^2^	0.254	0.241	0.399	0.389
KP-F			221.347	

Based on the above results, it can be concluded that the coefficient of environmental regulation on carbon emissions is statistically significant at the 1% level, and the sign of the coefficient is consistent with that of the baseline regression. These results further consolidate the empirical robustness of the significant inhibitory effect of environmental regulation on carbon emissions, supporting the validity of Hypothesis H_12_. Moreover, the estimates of other control variables are largely consistent with the baseline regression results, indicating that the baseline results pass the robustness test.

### Mechanism test

To test hypothesis H_21_ and H_22_, this paper constructs a mediating model that incorporates spatial effects, drawing on the methods of Zhu et al.^46^ and Baron et al.^48^.

14 $$Te{c_{it}}=\rho {W_{ij}}Te{c_{it}}+{\tau _0}E{R_{it}}+{\tau _1}{X_{it}}+{d_i}+{u_t}+{\mu _{it}}+{\varepsilon _{it}}$$

15 $$C{E_{it}}=\rho '{W_{ij}}C{E_{it}}+{\gamma _0}E{R_{it}}+{\gamma _1}{X_{it}}+{\gamma _2}Te{c_{it}}+{d_i}+{u_t}+{\mu _{it}}+{\varepsilon _{it}}$$

Where, $Te{c_{it}}$represents the technological level of city *i* in the *t*-th year; $E{R_{it}}$represents the environmental regulation level of city *i* in the *t*-th year; ${X_{it}}$represents the set of all control variables in this; $C{E_{it}}$represents the carbon emissions of city *i* in the *t*-th year; ${W_{ij}}$represents spatial matrix; $\rho$ and $\rho '$ represent the spatial correlation coefficients for technological level and carbon emissions respectively; ${\mu _{it}}$represents the spatial error term; ${\varepsilon _{it}}$represents a random error term. If the coefficient ${\tau _0}$ in Eq. (14) is significant to coefficient ${\gamma _2}$ in Eq. (15) both statistically significant, and the sign of the interaction term ${\tau _0} \times {\gamma _2}$ aligns with the sign of coefficient ${\gamma _0}$, it indicates that there is an mediating effect. Conversely, if the sign of the interaction term ${\tau _0} \times {\gamma _2}$is opposite to the sign of coefficient ${\gamma _0}$, it suggests the presence of a suppression effect. At the same time, if coefficient ${\gamma _0}$ in Eq. (15) is significant, then $Te{c_{it}}$ exerts a partial mediating effect; if coefficient ${\gamma _0}$ in Eq. (15) is not significant, then $Te{c_{it}}$ exerts a complete mediating effect.

First, a benchmark regression is conducted based on Eq. (8), with the results presented in column (1) of Table 7. Next, following Eq. (14), technological progress is incorporated as a mediating variable for regression analysis, with the results shown in column (2) of Table 7. Finally, based on Eq. (15), the mediating variable is included in the model for regression, and the results are presented in column (3) of Table 7.

**Table 7:** Mediating effects of technological progress.

	(1)	(2)	(3)
	CE	Tec	CE
ER	−0.209^***^	0.046^**^	−0.213^***^
	(−3.91)	(1.98)	(−3.99)
GDP	0.267^***^	0.138^**^	0.259^***^
	(10.10)	(2.18)	(9.79)
Cap	−0.025	0.005	0.028^*^
	(−1.59)	(0.14)	(−1.75)
Pop	−0.016	0.049	−0.019
	(−0.37)	(0.46)	(−0.43)
Fin	−0.138^***^	−0.028	−0.138^***^
	(−5.22)	(−0.44)	(−5.23)
Str	−0.163^***^	−0.253^***^	−0.151^***^
	(−4.23)	(−2.74)	(−3.91)
Lab	0.020^**^	0.084^***^	−0.017^**^
	(2.45)	(4.26)	(2.08)
Tec			−0.037^***^
			(−6.23)
$\rho$	2.239^***^	2.042^***^	2.238^***^
	(47.36)	(26.88)	(47.33)
City fixed effects	Yes	Yes	Yes
Year fixed effects	Yes	Yes	Yes
*N*	4896	4896	4896
*R* ^2^	0.239	0.638	0.247

In column (2) of Table 7, the regression coefficient for environmental regulation is significantly positive, indicating that environmental regulation has a significant positive effect on technological progress. In column (3) of Table 7, the regression coefficient for technological progress is significantly negative, suggesting that technological progress contributes to urban carbon emission reduction. Moreover, when the regression coefficient for environmental regulation in column (2) of Table 7 is multiplied by the regression coefficient of technological progress in column (3) of Table 7, the sign of interaction term is negative. The sign of this interaction term is consistent with the coefficient of environmental regulation on carbon emissions in column (3) of Table 7, indicating that technological advancement mediates the relationship between environmental regulation and carbon emissions. Moreover, since the effect of environmental regulation on carbon emissions in column (3) of Table 7 passes the significance test at the 1% level, it indicates that technological advancement exerts a partial mediating effect in the relationship between environmental regulation and carbon emissions. Based on these findings, this paper empirically validates the rationality of Hypothesis H_22_, demonstrating that environmental regulation can effectively reduce aggregate carbon emissions through the mediating mechanism of technological advancement, thereby confirming the validity of the “Porter Hypothesis”.

To test hypothesis H_31_ and H_32_, this paper constructs the following mediating model that incorporates spatial effects.

16 $$FD{I_{it}}=\rho {W_{ij}}FD{I_{it}}+{\theta _0}E{R_{it}}+{\theta _1}{X_{it}}+{d_i}+{u_t}+{\mu _{it}}+{\varepsilon _{it}}$$

17 $$C{E_{it}}=\rho '{W_{ij}}C{E_{it}}+{\chi _0}E{R_{it}}+{\chi _1}{X_{it}}+{\chi _2}FD{I_{it}}+{d_i}+{u_t}+{\mu _{it}}+{\varepsilon _{it}}$$

Where, $Fd{i_{it}}$ represents the level of foreign investment of city *i* in the *t*-th year, and other variables are unchanged. First, a baseline regression is performed based on Eq. (8), with the regression results presented in column (1) of Table 8. Next, following Eq. (16), foreign investment is treated as a mediating variable and included in the regression model, with the results shown in column (2) of Table 8. Finally, based on Eq. (17), the mediating variable is incorporated into the model for regression, with the results displayed in column (3) of Table 8.

**Table 8:** Mediating effect of foreign direct investment.

	(1)	(2)	(3)
	CE	FDI	CE
ER	−0.209^***^	0.120^**^	−0.209^***^
	(−3.91)	(2.56)	(−3.90)
GDP	0.267^***^	0.465^***^	0.271^***^
	(10.10)	(5.28)	(10.21)
Cap	−0.025	0.467^***^	−0.022
	(−1.59)	(8.67)	(−1.39)
Pop	−0.016	0.015	−0.016
	(−0.37)	(0.11)	(−0.37)
Fin	−0.138^***^	−0.135	−0.138^***^
	(−5.22)	(−1.53)	(−5.23)
Str	−0.163^***^	−0.265^**^	−0.165^***^
	(−4.23)	(−2.06)	(−4.28)
Lab	0.020^**^	0.139^***^	0.021^**^
	(2.45)	(5.07)	(2.56)
FDI			−0.007^*^
			(−1.80)
$\rho$	2.239^***^	2.018^***^	2.238^***^
	(47.36)	(23.26)	(47.20)
City fixed effects	Yes	Yes	Yes
Year fixed effects	Yes	Yes	Yes
*N*	4896	4896	4896
*R* ^2^	0.239	0.345	0.241

In column (2) of Table 8, the regression coefficient for environmental regulation is significantly positive, indicating that the level of environmental regulation has a significant positive impact on foreign investment. In column (3) of Table 8, the regression coefficient for foreign investment is significantly negative, suggesting that foreign investment has a significant inhibitive effect on carbon emissions. Moreover, when the regression coefficient for environmental regulation in column (2) is multiplied by the regression coefficient for foreign investment in column (3), the sign of interaction term is negative. This interaction term has the same sign as the coefficient for the effect of environmental regulation on carbon emissions in column (3), indicating that foreign investment mediates the relationship between environmental regulation and carbon emissions. Furthermore, since the impact of environmental regulation on carbon emissions in column (3) passes the significance test at the 1% level, foreign investment exhibits a partial mediating effect in the relationship between environmental regulation and carbon emissions. Based on these findings, this study validates the rationale of Hypothesis H_32_, suggesting that environmental regulation can reduce total carbon emissions through the mediating effect of foreign investment, which is consistent with the “Pollution Halo Hypothesis”. However, from a spatial perspective, Table 5 shows that the indirect effect of environmental regulation (ER) is significantly positive. This suggests that while stringent local environmental regulations effectively constrain pollution emissions within the region, they may also induce the relocation of polluting capital or enterprises to adjacent areas, thereby generating a spatial spillover consistent with the “pollution haven” effect. This phenomenon highlights the potential tension between environmental regulation and the spatial reallocation of capital. On one hand, stricter regulation can improve the quality of foreign direct investment (FDI) and enhance technology spillovers, thereby promoting local green development and carbon reduction. On the other hand, pollution-intensive and energy-consuming firms, seeking to avoid the rising costs of environmental compliance, may relocate to neighboring regions with relatively lax environmental standards, thereby increasing carbon emission pressures in those areas. These two forces are not inherently contradictory but rather interact to shape the spatial distribution of carbon emissions: local regions may exhibit a “pollution halo” effect, while adjacent areas may become “pollution havens.” This result underscores the need for policymakers to account for regional coordination in environmental governance. Strengthening local environmental regulation alone may not be sufficient; efforts must also be made to mitigate the “spatial leakage effect” caused by uneven regulatory intensity across regions. Future research should further explore the design of cross-regional regulatory coordination and joint pollution control mechanisms to facilitate the effective achievement of regional carbon reduction goals.

## Conclusions and recommendations

In the “new normal” stage, the Chinese environmental regulation model, characterized by government-led and market-assisted approaches, provides strong institutional support for promoting fundamental improvements in the ecological environment, advancing ecological civilization, and building a Beautiful China. It is crucial for China to explore long-term mechanisms and pathways through which environmental regulation can effectively promote carbon emission reductions. Based on panel data from 272 prefecture-level and above cities in China from 2003 to 2020, this study employs a composite indicator method to measure the intensity of urban environmental regulation, and analyzes the spatiotemporal distribution patterns and evolutionary trends of both total carbon emissions and environmental regulation intensity. Utilizing spatial autoregressive models and mediation effect models, this paper conducts an in-depth investigation into the impact of environmental regulation on carbon emissions and its transmission mechanisms. The study arrives at the following main conclusions: First, both the intensity of environmental regulation and carbon emissions across Chinese cities exhibit significant spatiotemporal heterogeneity. From a temporal perspective, environmental regulation levels and carbon emissions have both shown a fluctuating upward trend, with carbon emissions exhibiting more distinct stage characteristics. From a spatial perspective, environmental regulation intensity is generally lower in northern cities than in southern ones, while carbon emissions are more concentrated in North China, Northeast China, and western regions, and lower in the southern regions. Second, environmental regulation is shown to effectively suppress carbon emissions, thus validating the “forced emission reduction” hypothesis. Third, technological progress plays a partial mediating role in the relationship between environmental regulation and carbon emissions, thereby supporting the “Porter Hypothesis”. Fourth, foreign direct investment also serves as a partial mediator in this relationship, confirming the “pollution halo” hypothesis.

Based on theoretical analysis and empirical tests, this paper proposes the following policy recommendations: First, regional environmental regulation intensity should be strengthened, and differentiated regional environmental governance policies should be implemented. While environmental regulation policies can effectively promote carbon emission reductions, the spatiotemporal evolution patterns of carbon emissions and regulatory intensity indicate significant regional disparities, suggesting the need for context-specific, regionally differentiated regulatory policies to achieve low-carbon goals. In resource-based cities in northern China, environmental regulation intensity should be increased, outdated production capacity should be gradually phased out, and green industrial transformation should be promoted. In eastern coastal cities, current regulatory measures should be further strengthened, with an emphasis on refining and precisely targeting regulatory tools to consolidate green development achievements. Western regions should leverage national ecological governance policies to further enhance ecological compensation and regulatory incentives, thereby guiding green industrial upgrading. Second, by defining an appropriate range of regulatory intensity, firms can be reasonably incentivized to engage in technological innovation, thereby achieving coordinated progress in technological advancement and green development. Moderate regulation refers to a level of regulatory intensity that effectively constrains pollution emissions without suppressing corporate innovation. Regulation that is too lenient fails to stimulate innovation, while overly stringent regulation may crowd out firm investment and production. Regulatory intensity should therefore be set within a range that considers firms’ cost-bearing capacity, so as to pressure firms into adopting technological upgrades and pursuing green innovation. This in turn will promote the development of green industries and technologies, enable green industrial transformation, and advance regional carbon emission reductions. Third, the government should formulate targeted policies for foreign investment, strictly control the entry of pollution-intensive foreign capital, and encourage environmentally friendly foreign direct investment. Through the “pollution halo effect”, technology spillovers can be realized, thereby promoting regional low-carbon development. Simultaneously, environmental review standards for foreign-invested projects should be raised, and firms with low-carbon technologies and advanced environmental management practices should be actively selected. This approach will help avoid the relocation of pollution-intensive industries to regions with weak regulation, thereby enhancing the positive environmental effects of foreign capital and promoting the formation of a new low-carbon development model.

## Further discussion

The primary objective of this study is to examine how government-led environmental regulation influences urban carbon emission performance through spatial spillover effects and multidimensional transmission mechanisms. By identifying these operative pathways, the study seeks to establish scientifically grounded regulatory intensities and structurally optimized control strategies, thereby providing empirical evidence and policy insights to enhance urban green governance and promote coordinated regional carbon mitigation. Despite the systematic theoretical framework and rigorous empirical analysis presented, several limitations warrant further attention in future research. First, the carbon emission data utilized in this study are sourced from third-party institutions. While generally considered reliable and suitable for city-level analysis, potential estimation errors may arise due to methodological assumptions or limitations in data coverage, which could affect the precision of the empirical findings. Second, the construction of the spatial weight matrix inevitably involves a degree of subjectivity. While this study adopts an adjacency-based spatial weight matrix to capture the spatial spillover effects of environmental regulation, such a conventional approach may not fully account for substantive intercity linkages, including economic integration, industrial interdependence, and collaborative environmental governance. In particular, the absence of an economic-geographic spatial weight matrix that incorporates factors such as GDP differentials or intercity trade flows may constrain the ability to adequately capture the economic interdependencies among Chinese cities. Future research should therefore consider alternative weighting schemes, including economic-geographic matrices, to strengthen the robustness and validity of spatial effect identification. Third, the study does not explicitly differentiate between types of environmental regulation. It does not disaggregate policy instruments into market-based, command-and-control, and participatory categories, which may obscure differences in their mechanisms and outcomes. Future research could adopt policy text analysis and classification frameworks to systematically evaluate the transmission channels and effectiveness of different regulatory types, thereby enhancing the specificity and practical relevance of policy recommendations. Fourth, the scope of the mediation mechanism modeling is relatively narrow. Although the analysis incorporates green technological innovation and foreign direct investment as intermediary variables, it does not account for other potentially influential mechanisms such as industrial upgrading or changes in green consumption behavior. Subsequent research could expand the mediation framework by integrating variables related to industrial restructuring, energy transition, and consumer behavior, thereby offering a more comprehensive understanding of how environmental regulation affects carbon emissions. Fifth, the measurement of green technological innovation may be subject to identification bias. While the logarithmic transformation of “green invention patent applications plus one” is widely adopted and operationally convenient, it is susceptible to time lags and heterogeneity in patent quality, which may affect the accuracy of mediation effect estimates. The process from patent application to authorization and subsequent emission reduction typically involves a substantial delay, limiting the measure’s ability to capture short-term innovation effects. Furthermore, patent counts alone may not adequately reflect the quality or applicability of innovation, particularly in contexts where policy incentives lead to non-substantive patent filings. Future research should consider incorporating more refined indicators, such as the number of granted patents, firms’ green R&D investment, or green total factor productivity. Additionally, applying dynamic modeling approaches could more accurately represent the evolution and impact of technological progress.

In conclusion, future studies should aim to enhance data dimensionality, refine spatial modeling strategies, broaden the analytical scope of transmission mechanisms, differentiate regulatory instruments, and incorporate high-quality indicators alongside dynamic causal inference methods. These advancements will strengthen the scientific rigor and policy relevance of empirical research and offer more actionable insights to support the effective implementation of China’s “dual carbon” strategic objectives.

## Supplementary Information

Below is the link to the electronic supplementary material.

Supplementary Material 1

## Data Availability

All data generated or analysed during this study are included in this published article [and its supplementary information files].

## Declarations

### Explanation

The specific version of the ARCGIS software used for mapping in this study is ARCGIS 10.8.1.
